# The mediation and interaction of the obesity index between moderate-vigorous recreational physical activity and hypertension

**DOI:** 10.1371/journal.pone.0296333

**Published:** 2023-12-28

**Authors:** Bingqian Du, Yuting Li, Yun Xia, Shan Wu, Yuan Wei, Zhihao Wang, Shupei Wang, Jiao Guo, Qing Zhu, Tianran Shen, Qingsong Chen

**Affiliations:** 1 School of Public Health, Guangdong Pharmaceutical University, Guangzhou, China; 2 Guangdong Provincial Engineering Research Center of Public Health Detection and Assessment, Guangdong Pharmaceutical University, Guangzhou, China; 3 National Key Laboratory of Intelligent Tracking and Forecasting for Infectious Disease, National Institute for Communicable Disease Control and Prevention, Chinese Center for Disease Control and Prevention, Beijing, China; 4 Guangdong Metabolic Diseases Research Center of Integrated Chinese and Western Medicine, Guangzhou, China; 5 Key Laboratory of Glucolipid Metabolic Disorder, Guangzhou, China; 6 Institute of Chinese Medicine, Guangdong Pharmaceutical University, Guangzhou, China; 7 Guangdong TCM Key Laboratory for Metabolic Diseases, Guangzhou, China; Ajou University, REPUBLIC OF KOREA

## Abstract

Previous studies showed that physical activity (PA) is concerned with hypertension (HTN). However, the mediation and interaction role of the obesity index: body mass index (BMI), waist-hip ratio (WHR), body fat rate (BFR) and visceral fat index (VFI) between PA and HTN has never been studied. Therefore, the purpose of this study was to assess the mediation and interaction of the obesity index between moderate-vigorous recreational physical activity (MVRPA) and HTN. We conducted a cross-sectional study of 4710 individuals aged 41 or older in Torch Development Zone, Zhongshan City. The mediation and interaction of the obesity index were evaluated by a four-way decomposition. 48.07% of participants had HTN among these groups. In the adjusted linear regression model, MVRPA was significantly correlated with WHR (*β*±*SE* = -0.005±0.002; *P*<0.05). Compared to sufficient MVRPA (odds ratio (*OR*) = 1.35), 95% (confidence interval (*CI*) = 1.17–1.56), insufficient MVRPA increased the risk of developing HTN. Furthermore, there were associations between BMI, WHR, BFR, VFI and HTN where the adjusted *OR*s and 95% *CI*s were 1.11 (1.09–1.13), 6.23 (2.61–14.90), 1.04 (1.03–1.06), 1.07 (1.06–1.09), respectively. The mediation analyses suggested that the impact of MVRPA on HTN risk may partly be explained by changes in obesity index, with a pure indirect mediation of WHR between MVRPA and HTN (*P*<0.05). Therefore, weight control, especially reducing abdominal obesity and maintaining adequate MVRPA, may lead to more proper control of HTN.

## 1. Introduction

One quarter of adults worldwide are affected by hypertension (HTN), which is the main cause of all-cause mortality and a serious global public health problem [1, 2]. A survey in China showed that the incidence rate of HTN is very high, and about 44.7% of adults have HTN [3, 4]. The older adults in China is constantly increasing, and the government and healthcare institutions have conducted extensive research and intervention efforts to prevent the prevalence of HTN. However, the control of HTN is still not optimistic [5, 6]. Factors cannot be ignored are: the age of HTN patients is decreasing, and the use of convenient interventions is not realistic [7].

In recent decades, the mechanism by which conventional physical activity (PA) has a protective effect on HTN has been extensively studied [8–10]. A systematic review also pointed out that people who participate in a large amount of leisure-time PA have a lower HTN risk than those who participate in a low amount of leisure-time PA [11]. A possible mechanism is to change the obesity status of individual [12]. Obesity is usually simply defined as abnormal or excessive accumulation in adipose tissue [13]. Body mass index (BMI) can be used to estimate the prevalence and associated risks of obesity in the population, and it is the simplest obesity measurement method. However, BMI does not take into account the wide variation in the distribution of body fat, and obesity or its associated health risks do not necessarily correspond across individuals and groups. Significant changes in abdominal fat mass within a narrow range of systemic fat or BMI. In addition, it is also possible to identify the increased risk from obesity-related diseases due to abdominal fat accumulation by measuring waist-hip ratio (WHR), body fat rate (BFR) and visceral fat index (VFI). The 2010 Global Burden of Disease Study estimated that approximately 3.4 million people may die from overweight and obesity [14]. A study has found a nearly linear correlation between BMI and blood pressure (BP) [15]. The increase in VFI in obese individuals may also lead to an increase in BP levels [16]. These studies confirm the connection between PA and obesity index. However, there is limited research on the combined effects of PA and obesity index on HTN. The mediation and interaction effects of obesity indices such as BMI, WHR, BFR, and VFI between moderate-vigorous recreational physical activity (MVRPA) and HTN, as well as their combined effects, are still unclear. Therefore, it is necessary to conduct this study to understand the link between PA and HTN risks in response to the high prevalence of HTN.

The relationship between exposure and outcome can be explained through the concepts of mediation and interaction, which can help explain the impact of a reason on the "how" and "for whom" of a result [17]. In this study, the mediation hypothesis is that individuals with insufficient MVRPA may increase the risk of unhealthy obesity index, which in turn may increase the risk of HTN. According to the interaction hypothesis, the unhealthy obesity index may have a stronger correlation with the risk of HTN in individuals with insufficient MVRPA. But there may be both mediation and interaction. In this context, traditional mediation analysis cannot be effectively conducted and interpreted due to the inability to take into account or distinguish different factors. In our study, we use J VanderWeele’s four-way decomposition method performs mediation/interaction analysis, which divides mediation and interaction effects into four components: (i) the effect of the exposure in the absence of the mediator, (ii) the interactive effect when the mediator is left to what it would be in the absence of exposure, (iii) a mediated interaction (INT_med_), and (iv) a pure indirect effect (PIE) [18]. Compared to analyzing mediation and interaction effects separately, the four-way decomposition method can better reflect the correlation between exposure effects, mediation effects, and outcomes [19, 20].

The purpose of this study is to evaluate the mediation and interaction of the obesity index between MVRPA and HTN by applying a four-way decomposition method, so as to provide reference for the prevention and treatment of HTN.

## 2. Materials and methods

### 2.1 Study population

Our study was a cross-sectional survey used the baseline data from the Health Questionnaire of People with glucolipid metabolic disorder in Zhongshan City which is a predominantly development zone, and this study was based on the National Basic Public Health Service project. From May to October 2020, 11003 participants (aged≥41 years) enrolled at Zhongshan Torch Development Zone Hospital, Zhongshan City, Guangdong Province through local advertisements, invitation letters, health talks, or referrals from the local community. Individuals without severe infectious diseases, end-stage tumors, recent major surgeries, and external injuries who lived in the local area for more than 6 months were eligible to participate. Participants’ eligibility was verified by investigators in Zhongshan City. All eligible participants completed a self-administered questionnaire and participated in a physical examination which including health checkup (height, weight, waist circumference, hipline, BP, etc.), blood tests (fasting blood glucose (FBG), triglycerides). A standardized questionnaire and residents’ health records was used to assess demographic, anxiety, depression, and HTN. This study was conducted in accordance with the principles of the Declaration of Helsinki. This study has been approved by the Guangdong Pharmaceutical University Review Boards (Medical Ethics (2019) No.109). Paper informed consent was obtained from each participant, and the records were kept at Guangdong Pharmaceutical University.

**Fig 1:**
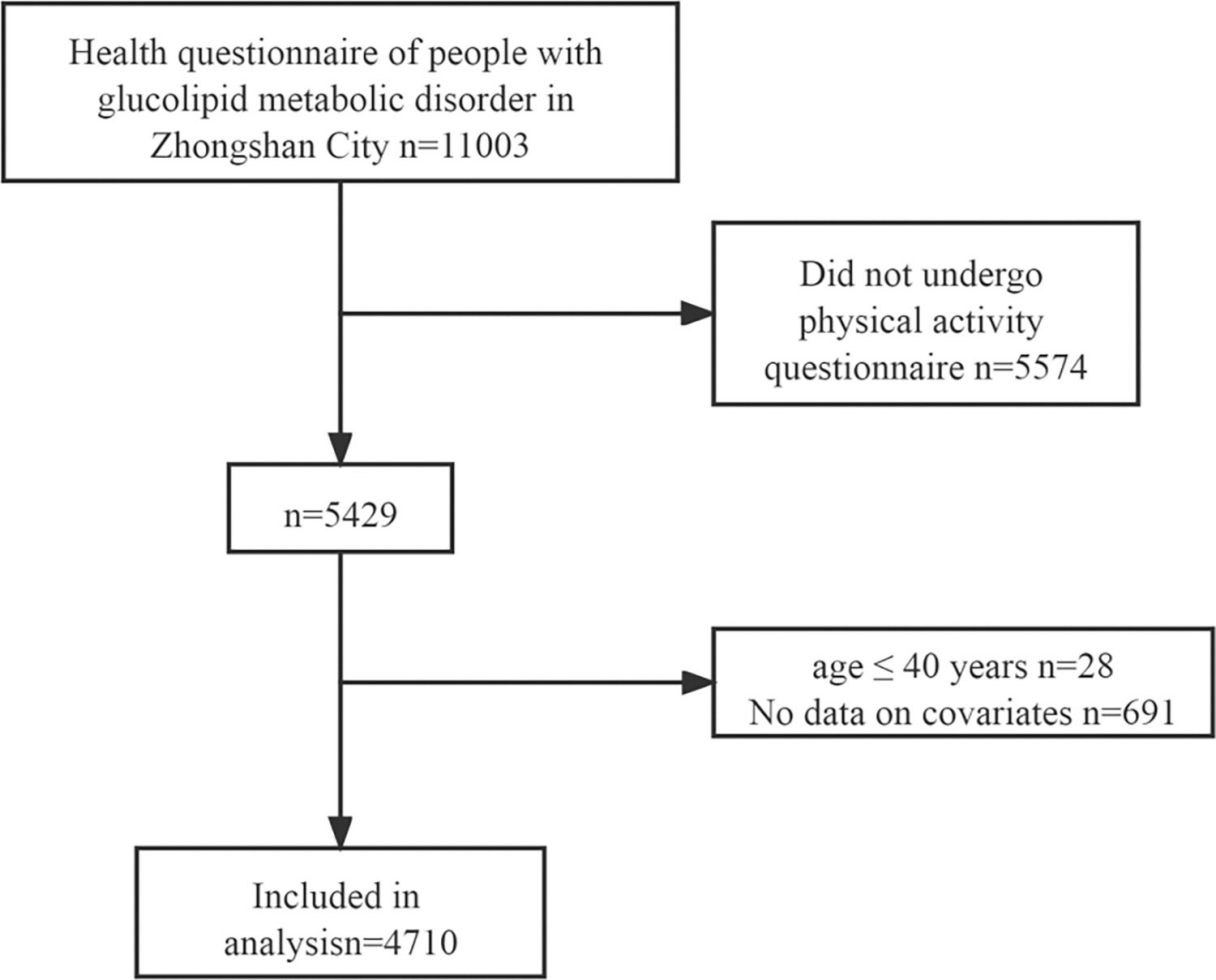
Flow chart of the study.

As shown in the flow chart (Fig 1), of the total 11003 participants, 5429 participants had participated in and fill out PA part of questionnaire. we excluded 28 individuals aged≤40 years, and 691 individuals with missing data of covariates or mediators (age, weight, height, waist circumference, hipline, family income, occupation, BFR and VFI). This left a total of 4710 participants (1173 men and 2937 women) for the final analyses.

### 2.2 Assessment of MVRPA

The PA section of the questionnaire is based on an abbreviated version of International Physical Activity Questionnaire (IPAQ) [21]. In this study, the types of MVRPA included moderate recreational physical activity (MRPA) and vigorous recreational physical activity (VRPA). Information on MVRPA was collected at enrollment. We define fitness or recreational activities that last for at least 10 minutes and result in a small increase in breathing or heart rate as MRPA (such as brisk walking, slow jogging, yoga, practising Tai Chi, cycling, climbing stairs and dancing). We define fitness or recreational activities that last for at least 10 minutes and result in a large increase in breathing or heart rate as VRPA (such as long-distance running, playing football, playing basketball, playing badminton, doing rope skipping and swimming). Sufficient MVRPA was defined as adults should undertake not less than 150 minutes of MRPA or 75 minutes of VRPA or an equivalent combination of MRPA and VRPA (1min of VRPA is equivalent to 2min of MRPA) per week according to WHO 2020 guidelines on PA and sedentary behaviour [22].

### 2.3 Assessment of HTN

For each participant, we measured BP three times on different days on their right upper arm after five min of rest in a seated position using an electronic sphygmomanometer (Omron HBP-1300, Omron Corporation, Dalian, China). HTN was defined by an average of three BP measurements with combining systolic BP (SBP)≥140 mmHg and/or diastolic BP (DBP)≥90 mmHg, or a self-reported diagnosis of HTN, or current use of antihypertensive medications [23].

### 2.4 Assessment of mediators

The height was measured by metal column height meter (TZG, Shkodak Medical, Shanghai, China), the unit of height is centimeter (cm), accurate to 0.1 cm. Weight, BMI, BFR, VFI were reported by body composition analyzer (Omron HBF-371, Omron Corporation, Beijing, China), the maximum weighing range is 140 kg, and the recorded results are accurate to 100 g. And all participants remained fasting for at least 8 hours before measurement. Waist circumference and hip circumference were measured by tape measure, and the figure is accurate to 0.1 cm. WHR was computed as waist circumference (cm) divided by hipline (cm) [24].

### 2.5 Assessment of other factors

Sociodemographic information included age (continuous), sex (male and female), nation (the Han nationality or others), education level (elementary education, junior high education, secondary general or vocational education, higher vocational education or university), occupation (civil servants and employees of firms and enterprises, professional and technical personnel, service works, workers and peasants or others), family income (≤¥4000, ¥4001-¥10000, >¥10000 or not quite clear), marital status (single people, married people, divorced people or widowed people), smoking habits (never smokers, ex-smokers or current smoker) and alcohol consumption (never drinkers, ex-drinkers or current drinkers). Depression was measured using the score from the Patient Health Questionnaire-9 (PHQ-9) [25]. Judgment score of standards for identifying possible depression is 5 or above [26]. Anxiety was measured using the score from the Generalized Anxiety Disorder Scale-7 (GAD-7). Judgment score of standards for identifying possible anxiety is 5 or above [27].

### 2.6 Statistical analyses

Baseline characteristics were calculated and displayed as median values (upper or lower quartile) for continuous variables and ratios for categorical variables. The comparison of categorical variables is conducted using chi-square test. Comparison of continuous variables using analysis of ANOVA analysis. Linear regression analyses were performed to assess the relationships between BMI, WHR, BFR, VFI and MVRPA. Logistic regression analyses were performed to identify (1) independent variables (MVRPA was treated as categorical variables) associated with HTN; (2) independent variables (BMI, WHR, BFR and VFI were treated as continuous variables) associated with HTN. 95% *CI* was obtained by Wald method. The covariates of the model were determined through literature review and correlation analysis with single factor analysis. Meanwhile, the continuous variable triglyceride and FBG with more information are included in the model rather than the disease. All models were adjusted for the following potential confounding factors: age, sex, nation, education level, occupation, family income, marital status, drinking, smoking, anxiety, depression, triglyceride and FBG.

To further analyze the role of obesity index in MVRPA and HTN, we use J VanderWeele’s four-way decomposition method performs mediation/interaction analysis, which divides mediation and interaction effects into four components: controlled direct effect (CDE), reference interaction (INT_ref_), INT_med_, and PIE [18]. The following four potential contributors are defined in this article:

- CDE: The effect of MVRPA has a direct effect on HTN.
- INT_ref_: This represents the impact on HTN that is due to the interaction between MVRPA and obesity index only.
- INT_med_: This represents the impact on HTN that is due to both mediation and interaction between MVRPA and obesity index.
- PIE: The effect of MVRPA on HTN which purely goes through WHR.

**Fig 2:**
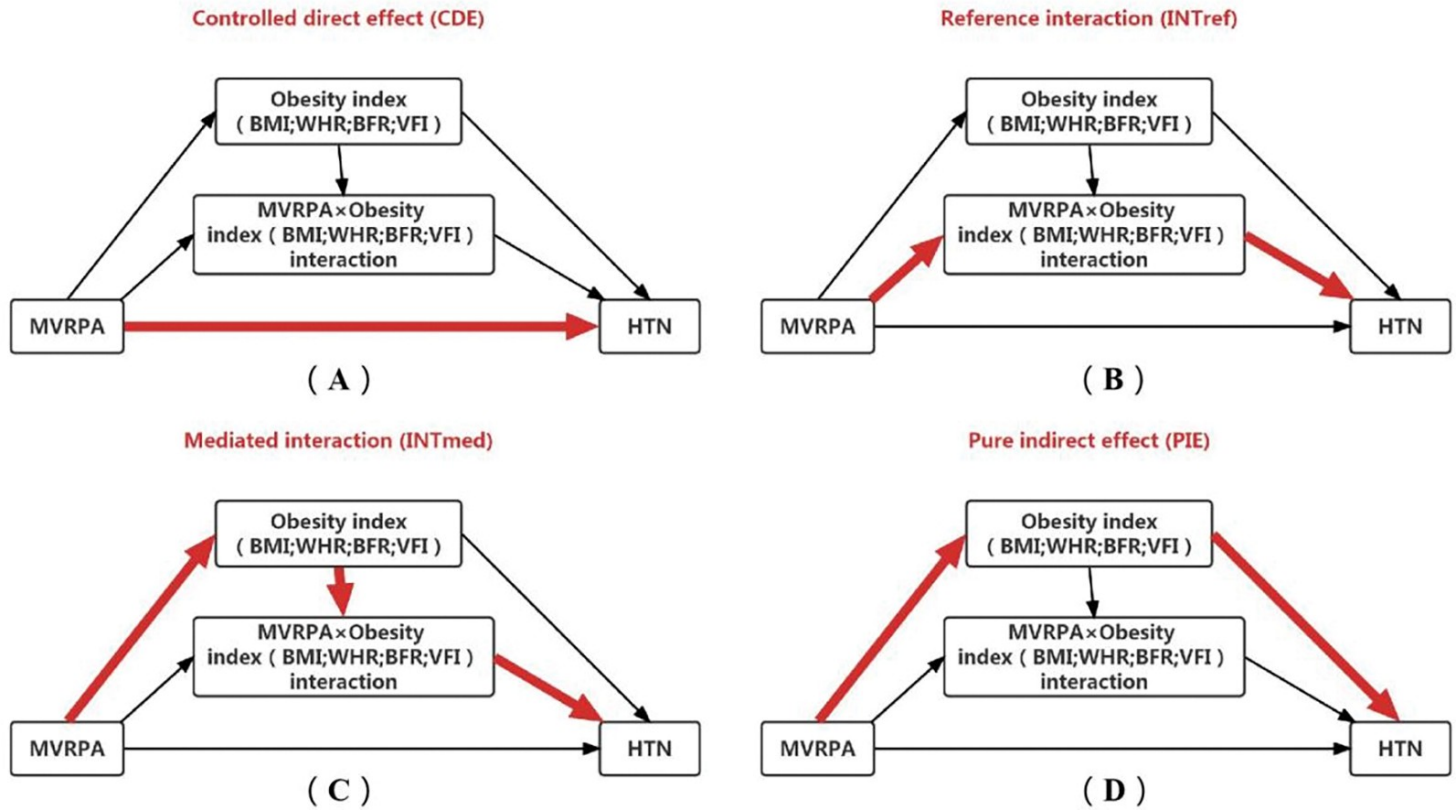
Summary of four-way decomposition in causal mediation analysis (note: Bolded arrows represent each of the four component of the decomposition). (A) CDE due to neither mediation nor interaction (in the current study: the effect of MVRPA has a direct effect on HTN), (B) INTref due to interaction alone, (C) INTmed due to both mediation and interaction, (D) PIE due to mediation alone (in the current study: the effect of MVRPA on HTN which purely goes through WHR).

Fig 2 shows a causal graphing representing the association of MVRPA with risk of HTN mediated by BMI, WHR, BFR and VFI. In SAS9.4, through the combination of four paths, all the effects and proportion attributes of the four combinations can be output. All analyses were performed with SAS version 9.4 software (SAS Institute Inc, Cary, NC, USA). Two-tailed *P*-values of <0.05 were considered statistically significant.

## 3. Results

Table 1 shows the baseline characteristics of the study population by sufficient MVRPA and insufficient MVRPA. Of the 4710 subjects, 2937 (62.4%) were female, 3578 (76.0%) had insufficient MVRPA. Compared with participants in the insufficient MVRPA group, those in the sufficient MVRPA group were less likely to be low education,smokers, depression, anxiety and HTN.

**Table 1:** Characteristics of the study population by sufficient MVRPA and insufficient MVRPA.

	Insufficient MVRPA	Sufficient MVRPA	*P* for difference
**N**	3578	1132	
**Gender**			0.011
Female	2195 (61.3)	742 (65.5)	
Male	1383 (38.7)	390 (34.5)	
**Age**	66.93±7.16	66.30±6.34	0.008
**Education**			<0.001
Elementary education	1828 (51.1)	439 (38.8)	
Junior high education	985 (27.5)	346 (30.6)	
Secondary general or vocational education	617 (17.3)	254 (22.4)	
Higher vocational education or university	148 (4.1)	93 (8.2)	
**Occupation**			<0.001
Civil servants and employees of firms and enterprises	452 (12.6)	191 (16.9)	
Professional and technical personnel	189 (5.3)	95 (8.4)	
Service works	537 (15.0)	167 (14.7)	
Workers and peasants	1847 (51.6)	499 (44.1)	
Others	553 (15.5)	180 (15.9)	
**Family income**			0.001
≤ ¥4000	2386(66.7)	713 (63.0)	
¥4001 ~ ¥10000	451 (12.6)	187 (16.5)	
> ¥10000	49 (1.4)	25 (2.2)	
Not quite clear	692 (19.3)	207 (18.3)	
**Marital status**			0.269
Single people	18 (0.5)	1 (0.1)	
Married people	2912 (81.4)	928 (82.0)	
Divorced people	67 (1.9)	27 (2.4)	
Widowed people	569 (15.9)	173 (15.2)	
Not quite clear	12 (0.3)	3 (0.3)	
**Smoking status**			0.003
Non-smoker	2773 (77.5)	916 (80.9)	
Smoker	486 (13.6)	110 (9.7)	
People who have quit smoking	319 (8.9)	106 (9.4)	
**Drinking situation**			0.915
Non-drinker	3209 (89.7)	1014 (89.6)	
Drinker	369 (10.3)	118 (10.4)	
**BMI**	23.95±3.27	23.75±3.24	0.064
**WHR**	0.91±0.07	0.90±0.07	<0.001
**BFR**	31.55±6.44	31.57±6.68	0.932
**VFI**	9.72±4.79	9.31±5.06	0.013
**Depression**			<0.001
No	3414 (95.4)	1105 (97.6)	
Yes	164 (4.6)	27 (2.4)	
**Anxiety**			<0.001
No	3293 (92.0)	1080 (95.4)	
Yes	285 (8.0)	52 (4.6)	
**HTN**			<0.001
No	1780 (49.7)	666 (58.8)	
Yes	1798 (50.3)	466 (41.2)	
**FBG**	5.81±1.89	5.65±1.61	0.014
**Triglyceride**	1.78±1.42	1.68±1.47	0.033

Note: Chi-square test was used for categorical variables; ANOVA was used for continuous variables. Results are expressed as mean differences ± standard deviation (Mean ± SD) or number (percent).

Table 2 shows the baseline characteristics of the study population by non-HTN and HTN. Of the 4710 subjects, 2264 (48.1%) participants had HTN. Compared with participants in the non-HTN group, those in the HTN group were more likely to be low education, without spouses.

**Table 2:** Characteristics of the study population by non-HTN and HTN.

	Non-HTN	HTN	*P* for difference
**N**	2446	2264	
**Gender**			0.707
Female	1519 (62.1)	1418 (62.6)	
Male	927 (37.9)	846 (37.4)	
**Age**	66.50±6.16	67.08±7.75	0.004
**Education**			<0.001
Elementary education	1116 (45.6)	1151 (50.8)	
Junior high education	680 (27.8)	651 (28.8)	
Secondary general or vocational education	508 (20.8)	363 (16.0)	
Higher vocational education or university	142 (5.8)	99 (4.4)	
**Occupation**			0.077
Civil servants and employees of firms and enterprises	364 (14.9)	279 (12.3)	
Professional and technical personnel	149 (6.1)	135 (6.0)	
Service works	353 (14.4)	351 (15.5)	
Workers and peasants	1190 (48.7)	1156 (51.1)	
Others	390 (15.9)	343 (15.2)	
**Family income**			0.164
≤ ¥4000	1583 (64.7)	1516 (67.0)	
¥4001 ~ ¥10000	355 (14.5)	283 (12.5)	
> ¥10000	42 (1.7)	32 (1.4)	
Not quite clear	466 (19.1)	433 (19.1)	
**Marital status**			0.001
Single people	7 (0.3)	12 (0.5)	
Married people	2048 (83.7)	1792 (79.2)	
Divorced people	46 (1.9)	48 (2.1)	
Widowed people	337 (13.8)	405 (17.9)	
Not quite clear	8 (0.3)	7 (0.3)	
**Smoking status**			0.272
Non-smoker	1905 (77.9)	1784 (78.8)	
Smoker	327 (13.4)	269 (11.9)	
People who have quit smoking	214 (8.7)	211 (9.3)	
**Drinking situation**			0.068
Non-drinker	2174 (88.9)	2049 (90.5)	
Drinker	272 (11.1)	215 (9.5)	
**BMI**	23.34±3.11	24.51±3.32	<0.001
**WHR**	0.91±0.07	0.92±0.07	<0.001
**BFR**	31.04±6.54	32.11±6.41	<0.001
**VFI**	8.94±4.66	10.36±4.95	<0.001
**Depression**			0.072
No	2359 (96.4)	2160 (95.4)	
Yes	81 (3.3)	104 (4.6)	
**Anxiety**			0.571
No	2276 (93.0)	2097 (92.6)	
Yes	170 (7.0)	167 (7.4)	
**FBG**	5.65±1.68	5.90±1.96	<0.001
**Triglyceride**	1.63±1.22	1.88±1.60	<0.001

Note: Chi-square test was used for categorical variables; ANOVA was used for continuous variables. Results are expressed as mean differences ± standard deviation (`Mean ± SD) or number (percent).

The linear regression revealed that MVRPA was associated with WHR and VFI before adjusted (*β*±*SE* = -0.010±0.002, -0.412±0.166, respectively; *P* <0.05) (Table 3). After adjustment for age, sex, nation, education, occupation, family income, marital status, drinking, smoking, and anxiety and depression, MVRPA still strongly associated with WHR (*β*±*SE* = -0.005±0.002; *P* <0.05). We did not observe the relation between MVRPA and BMI, BFR.

**Table 3:** Associations of MVRPA and obesity index in the study population (n = 4710).

	Unadjusted		Adjusted	
Dependent Variable	$\hat{\beta }$	*SE* (*β*)	$\hat{\beta }$	*SE* (*β*)
BMI	-0.206	0.111	-0.064	0.112
WHR	-0.010*	0.002	-0.005^†^	0.002
BFR	0.019	0.222	-0.144	0.160
VFI	-0.412^†^	0.166	-0.046	0.158

Note: Adjusted for age, sex, nation, education, occupation, family income, marital status, drinking, smoking, anxiety, depression, FBG and triglyceride.

*P<0.001

^†^*P<*0.05.

Table 4 shows that HTN was significantly associated with MVRPA before and after adjusting for potential confounders (crude *OR* = 1.44, 95%*CI* = 1.26–1.65; adjusted *OR* = 1.35, 95%*CI* = 1.17–1.56). Furthermore, there were associations between BMI, WHR, BFR, VFI and HTN where the multivariable-adjusted *OR*s and 95%*CI*s were 1.11 (1.09–1.13), 6.23 (2.61–14.90), 1.04 (1.03–1.06), 1.07 (1.06–1.09), respectively.

**Table 4:** Associations of HTN and MVRPA, BMI, WHR, BFR, VFI in the study population (n = 4710).

Variable	Crude OR (95%CI)	Adjusted OR (95%CI)
Insufficient MVRPA	1.44(1.26, 1.65)^†^	1.35 (1.17, 1.56)^†^
BMI	1.12 (1.10,1.14)*	1.11 (1.09, 1.13)*
WHR	12.62 (5.52, 28.84)*	6.23 (2.61, 14.90)*
BFR	1.03 (1.02, 1.04)*	1.04 (1.03, 1.06)*
VFI	1.07 (1.06, 1.09)*	1.07 (1.06, 1.09)*

Note: Adjusted for age, sex, nation, education, occupation, family income, marital status, drinking, smoking, anxiety, depression, FBG and triglyceride.

*P<0.001

^†^*P<*0.05.

The results of the four-way decomposition are shown in Table 5. In the model accounting for the presence of a total effect between MVRPA and obesity index (BMI, WHR, BFR and VFI) (*P*<0.001), insufficient MVRPA increases the risk of HTN: 1.39 (1.18–1.60), 1.35 (1.16–1.55), 1.38 (1.19–1.58), 1.33 (1.12–1.54), respectively. With BMI, BFR and VFI as being the mediator, we didn’t observe the mediation and / or interaction, the excess relative risk (*ERR*) and 95% *CI*s of CDE were 0.34 (0.16–0.53), 0.36(0.17–0.55), 0.33(0.15–0.51), respectively. A mediate effect of WHR between MVRPA and HTN was observed. Specifically, the *ERR* of the PIE generated by WHR is 0.02 (95% *CI*: 0.00–0.03), and 5% of the *ERR* of insufficient MVRPA on HTN compared to sufficient MVRPA is attributable to PIE. And the overall proportion mediated (PIE plus the INT_med_) by WHR was 4%.

**Table 5:** Four-way decomposition of the effect of MVRPA on HTN with BMI, WHR, BFR and VFI as the mediators in the study population.

	Current insufficient MVRPA compared to sufficient MVRPA Estimate (95%*CI*)			
	BMI	WHR	BFR	VFI
Total effect	1.39(1.18, 1.60)^†^	1.35(1.16, 1.55)^†^	1.38(1.19, 1.58)^†^	1.33(1.12, 1.54)^†^
*ERR* due to each component				
CDE	0.34(0.16, 0.53)^†^	0.35(0.16, 0.53)^†^	0.36(0.17, 0.55)^†^	0.33(0.15, 0.51)^†^
INT_ref_	0.03(-0.03, 0.08)	-0.01(-0.03, 0.01)	0.02(-0.01, 0.04)	-0.01(-0.07, 0.05)
INT_med_	0.01(-0.01, 0.02)	-0.00(-0.01, 0.01)	0.01(-0.01, 0.02)	0.00(-0.00, 0.01)
PIE	0.01(-0.01, 0.04)	0.02(0.00, 0.03)^†^	0.01(-0.01, 0.02)	0.01(-0.02, 0.04)
Proportion of effect due to each component				
CDE	88%(74%, 102%)^†^	97%(90%, 105%)^†^	93%(86%, 100%)^†^	100%(80%,121%)^†^
INT_ref_	7%(-5%, 20%)	-2%(-7%, 5%)	4%(-1%, 9%)	-3%(-22%, 16%)
INT_med_	2%(-2%, 5%)	-0%(-4%, 4%)	1%(-1%, 4%)	0%(-1%, 2%)
PIE	3%(-3%, 9%)	5%(-1%, 10%)	2%(-2%, 5%)	3%(-6%, 11%)
Overall proportion				
Mediated	5%(-4%, 13%)	4%(0%, 9%)^†^	3%(-3%, 8%)	3%(-6%, 12%)
Attributable to interaction	9%(-5%, 23%)	-2%(-11%, 8%)	5%(-1%, 11%)	-3%(-23%, 17%)
Eliminated	12%(-2%, 26%)	3%(-5%, 10%)	7%(0%, 14%) ^†^	-0%(-20%, 20%)

Note: Adjusted for age, sex, nation, education, occupation, family income, marital status, drinking, smoking, anxiety, depression, FBG and triglyceride.

^†^*P<*0.05.

## 4. Discussion

The results of the four-way decomposition analysis indicated that the effect of MVRPA on the risk of HTN may partly be explained by changes in obesity index, where there is PIE of WHR between MVRPA and HTN. The present study supplements the current literature regarding MVRPA and HTN among the elder population, and indicates WHR can more largely affect the association between MVRPA and the risk of HTN than BMI, BFR, and VFI.

According to our knowledge, there are few previous studies examining the interrelationships between MVPRA, obesity index and HTN, especially using the four-way decomposition method. Given that MVPRA is related to obesity index [28], our results indicated that the effects of MVPRA on HTN might be mediated by obesity index. Studies have shown that high levels of PA can reduce the risk of HTN by improving cardiovascular function and lowering levels of inflammatory factors [29, 30]. Furthermore, SBP, DBP and glycated hemoglobin (HbA1c) levels in people with high amounts of recreational sports and aerobic training were lower than non-exercising control group in middle-aged and older adults [31]. Our results are consistent with those findings. Similarly, a randomized controlled trial in older adults (66–80 years old) reported that home SBP (maximum between-group difference = 7.7 mmHg, *P* = 0.003) and home DBP (maximum between-group difference = 4.2 mmHg, *P* = 0.001) values of the exercise group is significantly lower than that of the non exercise group [32]. Recent WHO suggested that adults (18–64 years) and older adults (65 years and above) should, throughout the week, accumulate 150–300 minutes of moderate-intensity aerobic PA or 75–150 minutes of vigorous-intensity aerobic PA or an equivalent combination of moderate and vigorous PA [22]. Some studies also found that PA and all cause (CVD) cardiovascular disease mortality, cancer and diabetes incidence have a curvilinear dose-response relationship [22]. A China Health and Nutrition Survey 1991–2015 showed that the incidence rate of HTN increased from 10.86% in 1991 to 20.34% in 2015, while the median PA decreased from 408 MET·h/week to 104 MET·h/week at the same time. It is also pointed out that a high level of PA could to some extent alleviate the adverse effect of obesity index on HTN [33]. Moreover, a large national cohort study indicated that high BMI and low aerobic capacity in late adolescence were associated with higher risk of HTN in adulthood, and had a negative additive and multiplicative interaction [15]. However, we did not find a statistically significant interaction between PA and markers of obesity in present study. Decreasing PA and increasing BMI were associated with increased risk of HTN. Our findings consistent with Jackson’s study and suggest that both PA and maintenance of a healthy body weight are associated with lower risk of HTN [15], and there is a pure indirect mediation of WHR between MVRPA and HTN.

Participates with insufficient MVRPA have higher risk of HTN than those with sufficient MVRPA. In our study, only WHR as mediator has statistically significance PIE (4%, 0%-9%). This may due to WHR can reveal an unfavorable increase in abdominal fat much better than BMI, BFR and VFI [34]. Previous studies have shown that the deposition of abdominal fat organs is closely related to the occurrence of HTN [35, 36]. Previous research reports showed that abdominal obesity increases the likelihood of insulin resistance and elevated levels of non sterile fatty acid water, thereby increasing the risk of cardiovascular disease [37]. However, the mechanism by which determine fat distribution are not yet clear, and current data cannot explain why different behaviors are related to fat deposition at different locations. It has been suggested that visceral fat is particularly susceptible to adrenal-driven lipolysis during PA, which is why visceral fat is preferentially reduced in people with high PA levels [38]. Some potential biological mechanisms may explain how the impact of MVPRA on HTN is partially attributed to obesity index [39]. The findings showed that the reduction in PA-induced HTN was associated with post-exercise blood viscosity and aggregation of red blood cells [40]. At the same time, it can reduce sympathetic nerve tension, as to reduce the release of catecholamines, which reduce systemic vascular resistance [41, 42]. Indirectly, catecholamines act through β-adrenergic receptors in adipocytes, inducing an increase in cyclic adenosine monophosphate (cAMP) levels, which activates prekallikrein activator (PKA) and ultimately enhances lipolysis [43]. Adipose tissue is responsible for secreting leptin, a cytokine that boosts the activity of the sympathetic nervous system. So, lipolysis may lead to lower sympathetic nerve tension, relax peripheral artery, and lower BP [44].

This study has the following strengths: for the first time, the four-way decomposition method was used to explore the association between MVRPA and hypertension, but it has not been widely used in clinical research. Moreover, our study used a large, high compliance, baseline data of cohort population. Our study also has several limitations. First, we used a cross-sectional data, but the four-way decomposition may be more suitable for prospective cohort studies [19]. However, in our study, the causal sequence is basically reasonable given that HTN was regarded as chronic diseases, obesity index about BMI, WHR, BFR and VFI were all collected in hospital’s physical examination or residents’ health records. Second, the data of MVRPA are obtained from questionnaires, and it is inevitable that there will be errors when compared with the objective measurement results using accelerometers. Noticeably, the measure of MVRPA was based on the IPAQ with sufficient reliability and validity. Last, although we adjusted for study covariates in the analyses, there may be some unmeasured confounding factors in our study, such as participants’ eating habit, which is likely to impact PA behaviors and obesity index. Therefore, future studies are needed.

In conclusion, we found that in the predisposing factor of HTN, the effect of MVRPA on the risk of HTN may partly be explained by changes in WHR, where there is PIE of WHR between MVRPA and HTN. Therefore, weight control, especially reducing abdominal obesity and maintaining adequate MVRPA, may lead to more proper control of HTN. And our study may provide insights into the predisposing factor of clinical outcomes by causal mediation analysis in clinical research.
